# Hydrogen sulfide-mitigated salinity stress impact in sunflower seedlings was associated with improved photosynthesis performance and osmoregulation

**DOI:** 10.1186/s12870-024-05071-y

**Published:** 2024-05-18

**Authors:** Abeer Abdelrazk Younis, Mohamed Magdy Fahim Mansour

**Affiliations:** Faculty of Science, Department of Botany, Ain Shams University, Cairo, 11566 Egypt

**Keywords:** Chloroplast, Hydrogen sulfide, Photosynthesis, Rubisco, Salinity, Sunflower

## Abstract

**Background:**

Salinity is one major abiotic stress affecting photosynthesis, plant growth, and development, resulting in low-input crops. Although photosynthesis underlies the substantial productivity and biomass storage of crop yield, the response of the sunflower photosynthetic machinery to salinity imposition and how H_2_S mitigates the salinity-induced photosynthetic injury remains largely unclear. Seed priming with 0.5 mM NaHS, as a donor of H_2_S, was adopted to analyze this issue under NaCl stress. Primed and nonprime seeds were established in nonsaline soil irrigated with tape water for 14 d, and then the seedlings were exposed to 150 mM NaCl for 7 d under controlled growth conditions.

**Results:**

Salinity stress significantly harmed plant growth, photosynthetic parameters, the structural integrity of chloroplasts, and mesophyll cells. H_2_S priming improved the growth parameters, relative water content, stomatal density and aperture, photosynthetic pigments, photochemical efficiency of PSII, photosynthetic performance, soluble sugar as well as soluble protein contents while reducing proline and ABA under salinity. H_2_S also boosted the transcriptional level of ribulose 1,5-bisphosphate carboxylase small subunit gene (*HaRBCS*). Further, the transmission electron microscope showed that under H_2_S priming and salinity stress, mesophyll cells maintained their cell membrane integrity and integrated chloroplasts with well-developed thylakoid membranes.

**Conclusion:**

The results underscore the importance of H_2_S priming in maintaining photochemical efficiency, Rubisco activity, and preserving the chloroplast structure which participates in salinity stress adaptation, and possibly sunflower productivity under salinity imposition. This underpins retaining and minimizing the injury to the photosynthetic machinery to be a crucial trait in response of sunflower to salinity stress.

## Background

Among numerous abiotic stresses, salinity stress is a major abiotic constraint threatening global food security by decreasing agricultural productivity and a major hurdle in accomplishing the “zero hunger” goal proposed by FAO-UN [1]. Salinity stress is drastically increasing due to anthropogenic activities (i.e., irrigation malpractice, fertilization…etc.) and natural causes like soil salinization and poor water quality [2, 3]. Approximately 1.5 million hectares of farmland representing around 33% of irrigated land have been affected by salinity [4] and are predicted to climb up to a staggering 50% by 2050 [5]. Salinity stress involves ionic and osmotic stresses: osmotic stress is the first stress experienced when a plant is exposed to saline soil while ionic stress occurs later when salt levels reach a threshold beyond which the plant cannot maintain ion homeostasis and growth [6]. Both osmotic and ionic action of salinity ultimately cause oxidative stress affecting various physiological and biochemical processes in plants [7, 8], which eventually drastically impacts overall plant growth, development, metabolism, and productivity [9]. In addition, the changing climatic conditions and their effects on farming worsen the salinity problem and further threaten the stability of agricultural production. Therefore, addressing the salinity problem will be necessary for the future, given the growing population of the planet and the ever-increasing demand for food, which necessitate developing salinity-resilient crops required for sustainable agriculture and global food security.

Photosynthesis is the major source of energy that has significant implications on the redox status of plant cells and hence regulates all aspects of plant metabolism and physiology [10]. Therefore, the evaluation of the pivotal role of photosynthesis in plant phenotyping under stressful conditions is a very important task. Sarabi et al. [11] report that salinity stress exerts detrimental effects on photosynthesis, manifested through stomatal and non-stomatal limitations. Stomatal limitation refers to stomatal closure resulting in inefficient gas exchange and reduced photosynthetic rate [12, 13]. Non-stomatal limitations, mainly associated with the degradation of pigment-protein complexes, destruction of fine thylakoid membrane structure [14], reduction in PSII photochemical efficiency (F_v_/F_m_) [15], and decreased activity of ribulose 1,5-bisphosphate carboxylase/oxygenase (Rubisco) [16, 17]. Plant photosynthetic efficiency has been reported to be a reliable, non-destructive biomarker for determining the salinity tolerance of wheat genotypes [18]. It is also reported that the expression of the small subunit regulates the size of the Rubisco pool in plants and can influence the overall catalytic efficiency of the Rubisco complex [19]. Additionally, chlorophyll and carotenoids play a fundamental role in photosynthesis by capturing light energy and inducing electron transfer, and changes in their levels are related to the efficiency of the photosynthetic machinery under salinity stress [20, 21]. Therefore, the analysis of plant photosynthetic efficiency based on measurements of various photosynthetic parameters (photosynthetic performance (PI_abs_), the maximal quantum yield of PSII photochemistry (F_v_/F_m_), Chlorophyll a, chlorophyll b, carotenoids, and Rubisco small subunit expression) is an accurate approach for evaluating plant responses to unfavorable photosynthesis environmental conditions and their impact on crop plants [11, 17, 22].

Sunflower (*Helianthus annuus* L.) has great importance in the world economy as one of the most cultivated oilseed crops; the seed oil content ranges between 38 and 50% and is widely used in human food and the production of biodiesel [23, 24]. Also, sunflower seeds are a rich nutritional source, boasting high levels of protein, fiber, minerals, and phenolic compounds [25]. The cultivated area of sunflower in Egypt is tiny, although edible oil production is still deficient for population demand, with only 5% of the total oil demand covered by domestic production [26]. Therefore, increasing the domestic oil yield of sunflower is in demand and could be adopted by increasing the cultivated area, which may impose the use of saline soils, parallel to exploring the strategies for enhancing salinity tolerance of sunflower and promoting its production under this adverse conditions [26]. One of the short-term and most pragmatic approaches to boosting plant resistance to salinity is seed priming. Seed priming is a cost-effective eco-friendly technique that might have an enormous impact on oilseed production and sustainable agriculture and food security problems [27–29]. H_2_S, an emerging gaseous signaling molecule in plants, plays a pivotal role in regulating various physiological, biochemical, and developmental processes, including seed germination, plant growth, and development, regulates stomatal apertures, promotes photosynthetic activity, enhances plant’s resistance to abiotic stresses [30–33]. H_2_S as a signaling molecule in plants regulates these processes through crosstalk with other signaling molecules resulting in the repairing of biomembranes and denatured proteins as well under stress [34]. As a result, we hypothesized that the H_2_S priming to sunflower seeds could boost stomatal mechanics and protect the stability of chloroplast structure and photosynthetic pigments, which in turn, could enhance photosynthetic efficiency. Therefore, seed priming with NaHS (H_2_S donor) was adopted to analyze this approach’s efficiency in overcoming the negative adversities of salinity stress on sunflower photosynthesis. This is because how H_2_S stimulates sunflower salinity resilience in terms of photosynthetic apparatus performance remains largely unclear. To answer this question, we investigated the impact of H_2_S priming on photosynthetic parameters, Rubisco small subunit gene expression, chloroplast ultrastructure, and stomatal movement in sunflower seedlings under salinity stress, aiming to provide insights into its potential mitigation role on photosynthetic machinery of NaCl-stressed sunflower seedlings. This might participate in improving sunflower adaptation to salinity stress, and hence productivity under salinity imposition.

## Results

**Fig. 1:**
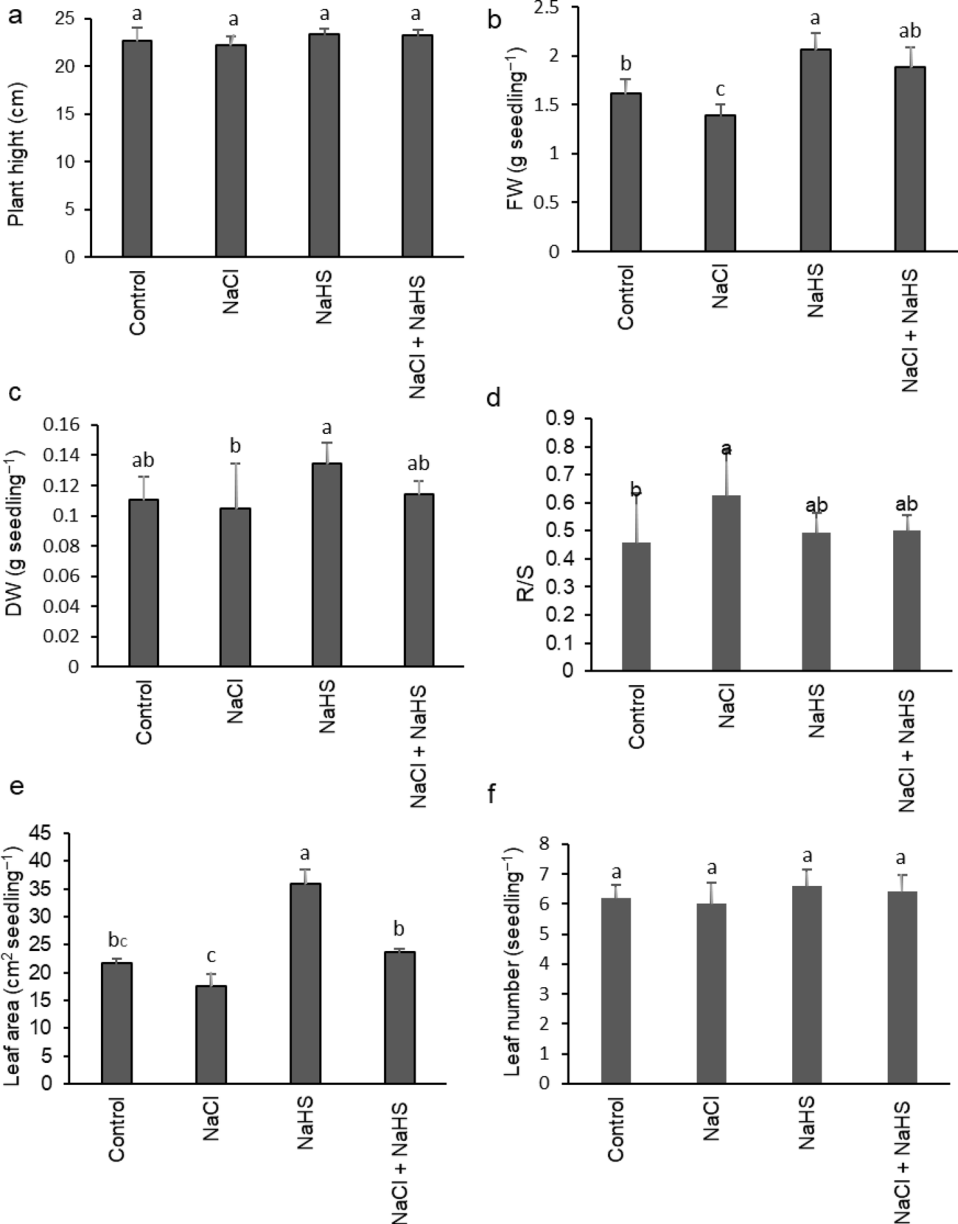
Effect of NaHS priming in the presence and absence of 150 mM NaCl on (**a**) plant height, (**b**) fresh weight (FW), (**c**) dry weight (DW), (**d**) root/shoot ratio (R/S), (**e**) leaf area, and (**f**) leaf number of sunflower seedlings. Each value is the mean ± SD of three replicates. Bars with different letters indicate significant differences at *P* ≤ 0.05

NaCl stress reduced sunflower seedlings’ growth in terms of plant fresh weight, dry weight, and leaf area, while increasing the root-to-shoot ratio (R/S ratio) (Fig. 1b-e). However, H_2_S priming significantly enhanced the growth of NaCl-stressed and non-stressed sunflower seedlings and reduced the R/S ratio in stressed seedlings relative to their corresponding controls (Fig. 1b-e). There is no significant difference in plant height and leaf number among treatments (Fig. 1a, f).

**Fig. 2:**
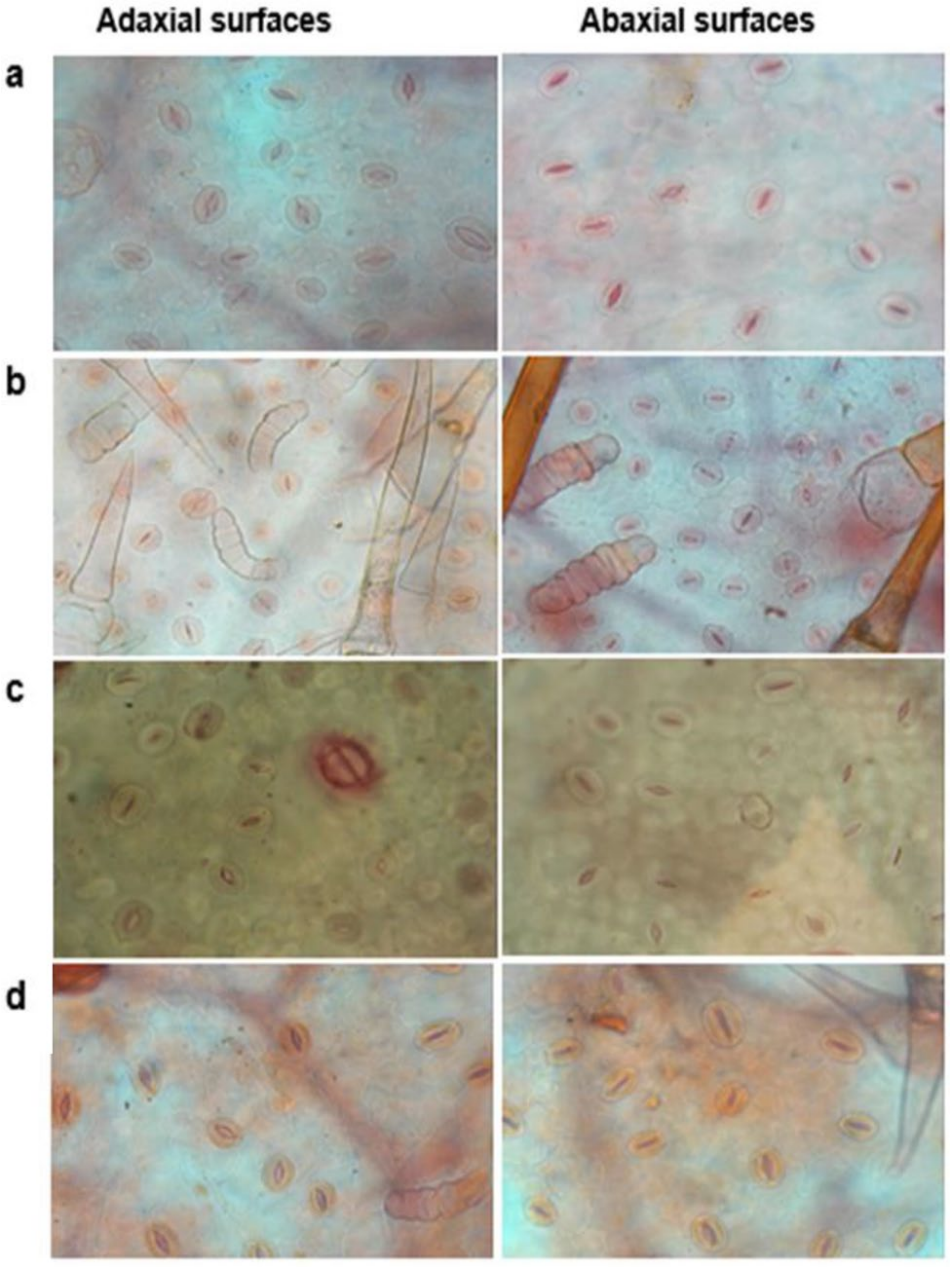
Effect of NaHS priming on stomatal characteristics of sunflower seedlings leaves in the presence and absence of 150 mM NaCl. (**a**) control, (**b**) NaCl, (**c**) NaSH, and (**d**) NaCl + NaSH

For stomatal characteristics, normal epidermal cells with stomata exhibited proper shape and open aperture in both adaxial and abaxial surfaces were observed in control and H_2_S alone treatments, (Fig. 2a, c). However, leaves of salinity-treated seedlings showed deformed guard cells, shrink epidermal cells, decreased stomatal size, closed apertures, as well as the presence of trichomes of thorn-like outgrowths (Fig. 2b). Plants received H_2_S priming and NaCl stress showed relatively normal shaped guard cells with partially opened stomatal aperture (Fig. 2d). Data presented in Table 1 revealed that several epidermis and stomata of salinity-stressed sunflower leaves significantly decreased by 22.5% and 22.3% for the adaxial surface and by 26.9% and 29.6% for the abaxial surface, respectively, compared with the control plants. Stomatal indices of adaxial and abaxial surfaces of salinity-stressed leaves significantly reduced by 26.2% and 20.7%, respectively, relative to the control ones (Table 1). Also, the reduced stomatal width and length in the salinity-treated plants resulted in a significantly reduced stomatal area of adaxial and abaxial surfaces (Table 1). The highest decrease in the stomatal area was observed on the abaxial surface (40%) of salinity-stressed leaves compared with the control (Table 1). On the other hand, H_2_S priming alleviated salinity-induced reductions in stomatal number, sizes, and stomatal indices of both surfaces of stressed leaves. The H_2_S-positive impacts were also observed in the non-stressed leaves (Table 1).

**Table 1:** The structure of the adaxial and abaxial epidermis in leaves of sunflower seedlings pretreated with NaHS, donor of H_2_S, in the presence and absence of 150 mM NaCl

	Treatment	Epidermal cells number (mm^2^)	Stomatal number (mm^2^)	Stomatal index	Stomatal length (µm)	Stomatal width (µm)	Stomatal area (µm^2^)
Adaxial surfaces	Control	107.50 ± 2.17^b^	37.33 ± 1.39^a^	25.77 ± 0.77^a^	20.30 ± 0.2^ab^	1.80 ± 0.14^b^	29.0 ± 1.67^b^
	NaCl	83.33 ± 0.64^d^	29.0 ± 0.97^c^	19.03 ± 0.64^c^	15.70 ± 0.15^c^	1.10 ± 0.10^d^	19.0 ± 1.44^d^
	NaHS	111.33 ± 3.16^a^	37.0 ± 1.03^ab^	25.15 ± 0.53^ab^	22.90 ± 0.35^a^	1.90 ± 0.2^a^	33.0 ± 1.89^a^
	NaCl + NaHS	96.66 ± 1.77^c^	34.33 ± 1.3^b^	23.0 ± 0.35^b^	19.60 ± 0.18^b^	1.30 ± 0.17^c^	24.0 ± 1.52^c^
Abaxial surfaces	Control	99.16 ± 1.18^b^	56.33 ± 1.59^ab^	36.23 ± 0.98^ab^	18.90 ± 0.18^ab^	1.10 ± 0.02^b^	15 ± 0.86^ab^
	NaCl	72.50 ± 1.2^d^	39.66 ± 1.34^c^	28.72 ± 0.88^c^	13.50 ± 0.13^c^	0.80 ± 0.03^d^	9.0 ± 0.62^c^
	NaHS	105.0 ± 1.97^a^	57.66 ± 1.2^a^	36.39 ± 1.03^a^	19.7 ± 0.17^a^	1.20 ± 0.11^a^	16.0 ± 0.95^a^
	NaCl + NaHS	81.66 ± 1.53^c^	47.33 ± 1.4^b^	32.05 ± 1.14^b^	17.7 ± 0.15^b^	0.90 ± 0.02^c^	12.0 ± 0.79^b^

Data with different superscript letters were significantly different (*P* ≤ 0.05). Data were expressed by mean values ± SE (*n* = 10)

**Fig. 3:**
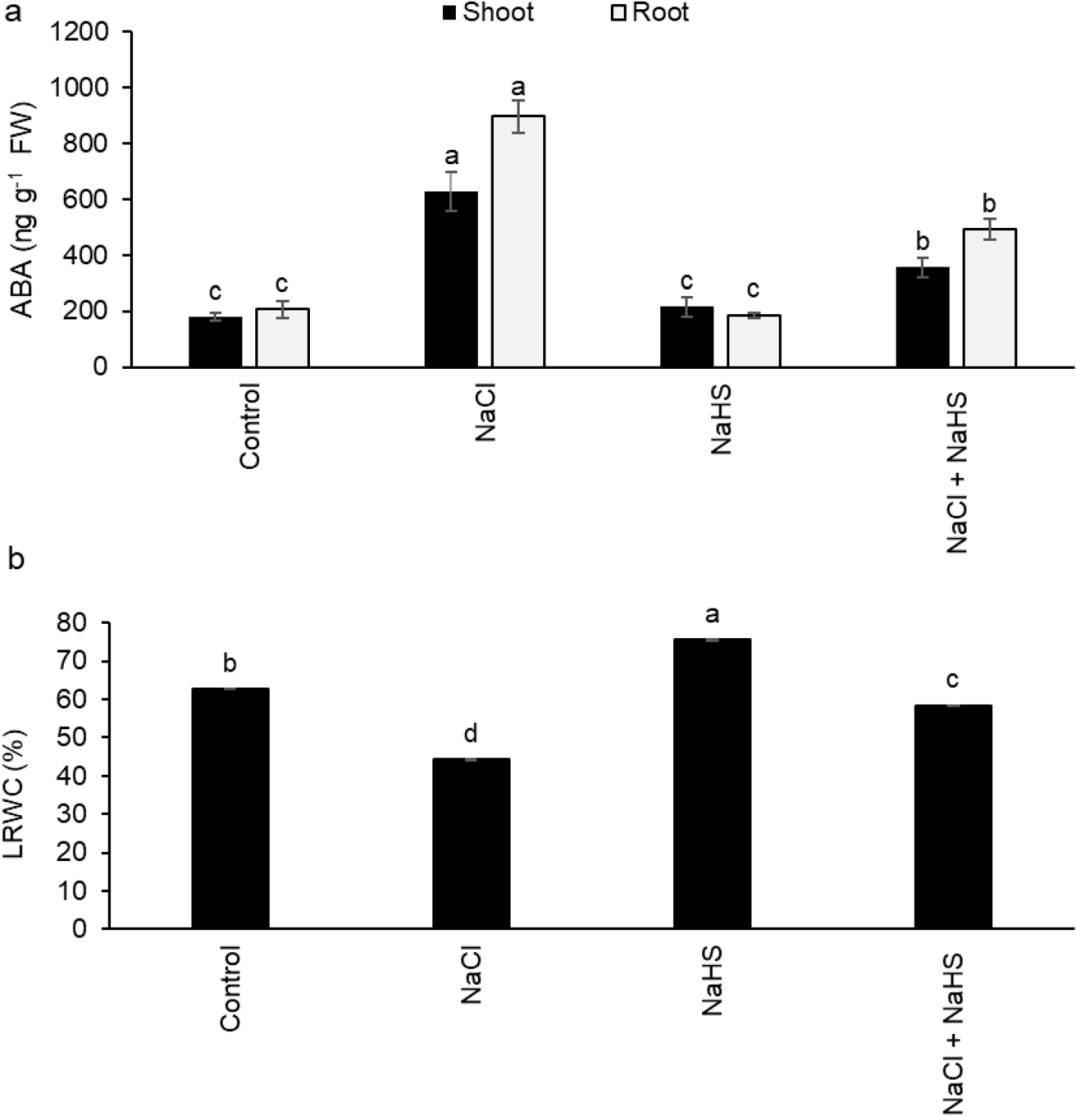
Effect of NaHS pretreatment in the presence and absence of 150 mM NaCl on: (**a**) ABA content and (**b**) leaf relative water content (LRWC) of sunflower seedlings. Each value is the mean ± SD of three replicates. Bars with different letters indicate significant differences at *P* ≤ 0.05

Exposure of sunflower seedlings to NaCl stress significantly increased the content of ABA by 248.7% and 335.6% in the shoots and roots, respectively, relative to their controls (Fig. 3a). H_2_S priming significantly reduced ABA content by 43.5% and 45.1% in the shoots and roots, respectively, compared with those received only NaCl stress (Fig. 3a). Salinity stress also remarkably decreased the LRWC by 29.3% in salinity-stressed seedlings relative to unstressed ones, whereas H_2_S priming significantly restored LRWC to the control value or even higher in stressed and non-stressed conditions (Fig. 3b).

**Fig. 4:**
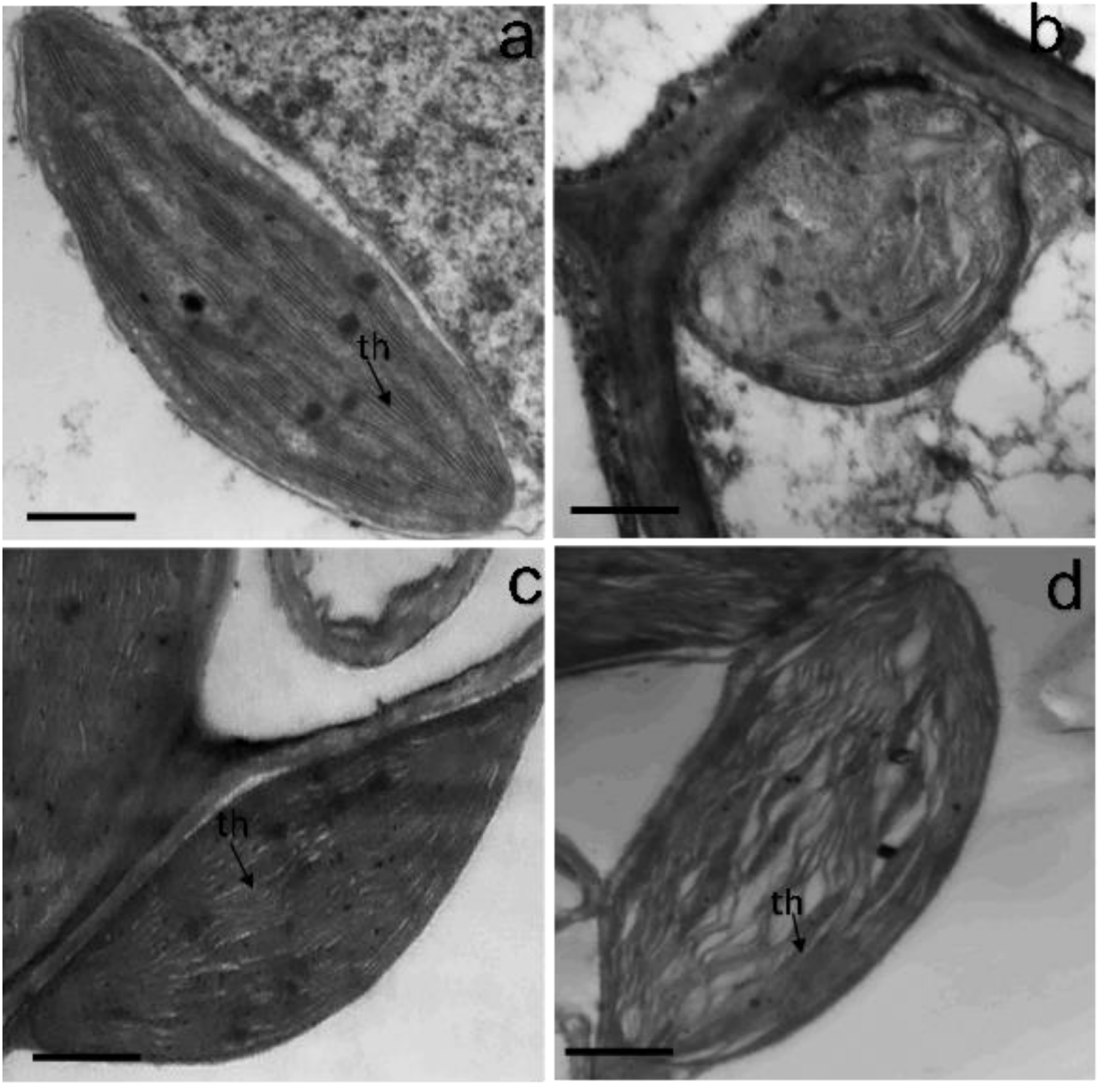
Effect of NaSH pretreatment on chloroplast ultrastructure of sunflower seedlings leaves in the presence and absence of 150 mM NaCl. (**a**) control, (**b**) NaCl, (**c**) NaSH, (**d**) NaCl + NaSH. th, thylakoid membranes. Bars are 500 nm

The chloroplast ultrastructure of the control and H_2_S-primed leaves exhibited ellipsoid shape with compactly arranged thylakoids and well-compartmentalized grana stacks with distinct grana lamellae (Fig. 4a, c), while under salinity stress, the chloroplast appeared rounded and displayed a disrupted envelope with a disorganized outer membrane, and loosened grana lamella with distorted thylakoid (Fig. 4b). However, H_2_S pretreatment alleviated the above salinity-induced ultrastructure disorders as distinct chloroplast membrane envelope, and a more regular arrangement of stroma thylakoids were observed compared with the samples received only NaCl stress (Fig. 4d).

**Fig. 5:**
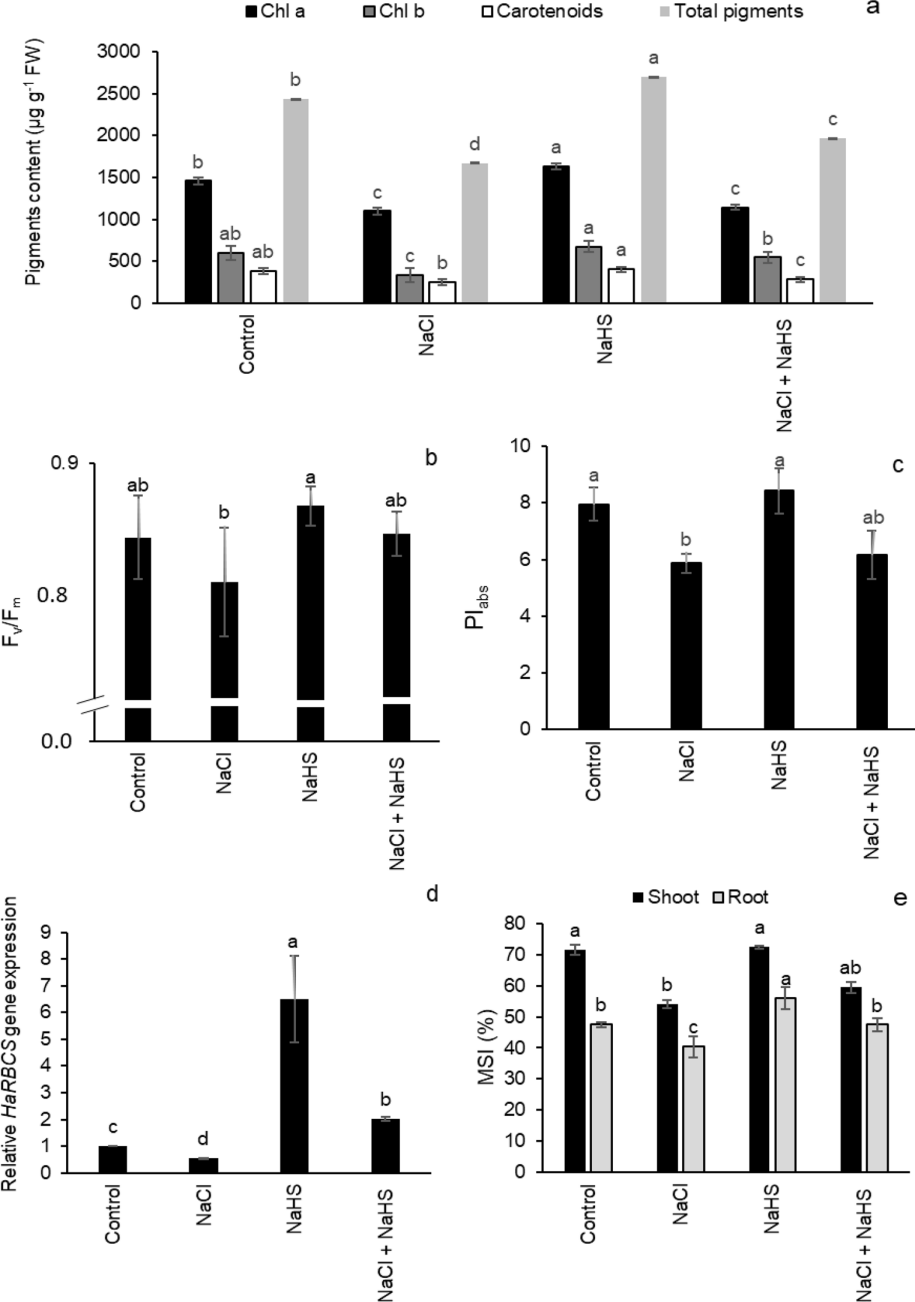
Effect of NaHS pretreatment in the presence and absence of 150 mM NaCl on (**a**) photosynthetic pigment contents, (**b**) maximum efficiency of photosystem II (F_v_/F_m_), (**c**) performance index (PI_abs_), (**d**) Rubisco small subunit (*HaRBCS*) gene expression, and (**e**) membrane stability index (MSI) of sunflower seedlings. Each value is the mean ± SD of three replicates. Bars with different letters indicate significant differences at *P* ≤ 0.05

The effect of 0.5 mM H_2_S priming on photosynthetic parameters in the leaves of sunflower seedlings under salinity stress is shown in Fig. 5. A significant decrease was observed in chlorophyll a, chlorophyll b, carotenoids, and total pigments under NaCl stress by 24.8%, 45%, 35%, and 31.3%, respectively, compared with the controls (Fig. 5a). H_2_S priming resulted in a significant increase in the photosynthetic pigment contents when it was applied alone or in the presence of NaCl stress compared with the NaCl controls (Fig. 5a). Further, NaCl stress induced a reduction in F_v_/F_m_ and PI_abs_ values, which were significantly restored to the control values or even higher with H_2_S priming (Fig. 5b, c). Also, the expression of the Rubisco small subunit gene *HaRBCS* was downregulated by 46% in response to NaCl treatment compared with the control, while H_2_S pretreatment with or without NaCl stress upregulated the expression of *HaRBCS* by 6.5 and 2.7 times, respectively, higher than that in the absence and presence of NaCl treatment (Fig. 5d). Salinity stress remarkably decreased the values of MSI in the seedling shoots and roots relative to unstressed ones; however, H_2_S priming significantly increased MSI in both stressed and non-stressed conditions (Fig. 5e).

**Fig. 6:**
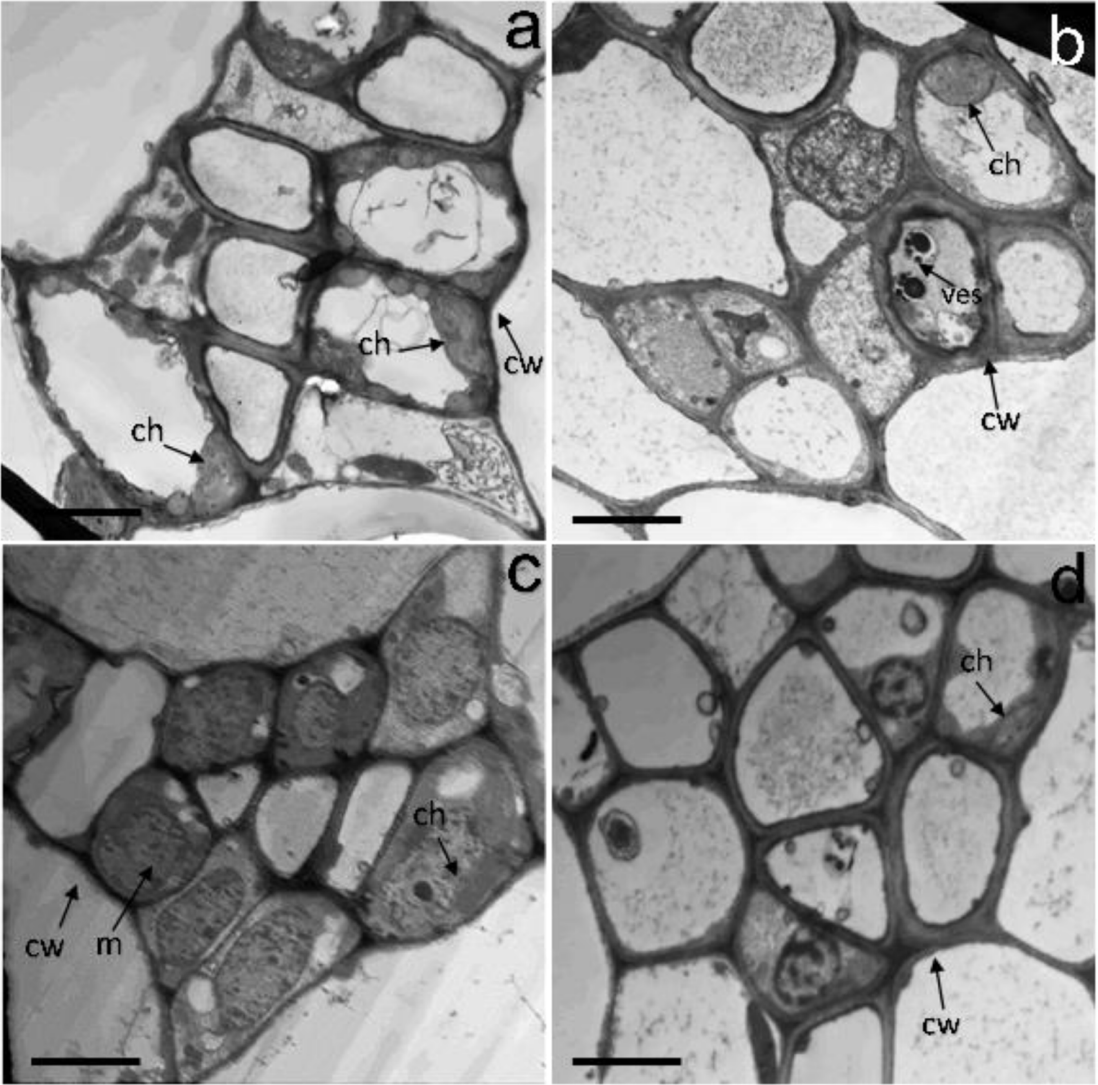
Effect of NaSH pretreatment on mesophyll cells ultrastructure of sunflower seedlings leaves in the presence and absence of 150 mM NaCl. (**a**) control, (**b**) NaCl, (**c**) NaSH, (**d**) NaCl + NaSH. cw, cell wall; ch) chloroplast; m) mitochondria; and ves) vesicles. Bars are 2 μm

The ultrastructure of the leaf mesophyll cells of the control or H_2_S-treated samples showed that all the organelles in the mesophyll cells were remarkably differentiated and well-developed (Fig. 6a, c). The cell membranes were also intact and in close contact with the cell wall, and the chloroplasts were closely arranged along the plasma membranes (Fig. 6a, c). Conversely, the fine structures of NaCl-treated leaves demonstrated partial plasmolysis in some cells, which was accompanied by a reduction in mesophyll intercellular spaces and complex vesiculation in the vacuoles and distortion of the nucleus (Fig. 6b). In addition, the number of chloroplasts decreased dramatically and showed disintegrated thylakoid membranes, and the mitochondria appeared also with dissolved cristae (Fig. 6b). On the other hand, the mesophyll cells structure under combined treatment of H_2_S and 150 mM NaCl showed less deleterious symptoms of NaCl toxicity as no shrinkage of the cell membranes observed, the chloroplasts retained their membrane envelopes, as well as more orderly mitochondria and nucleus (Fig. 6d).

**Fig. 7:**
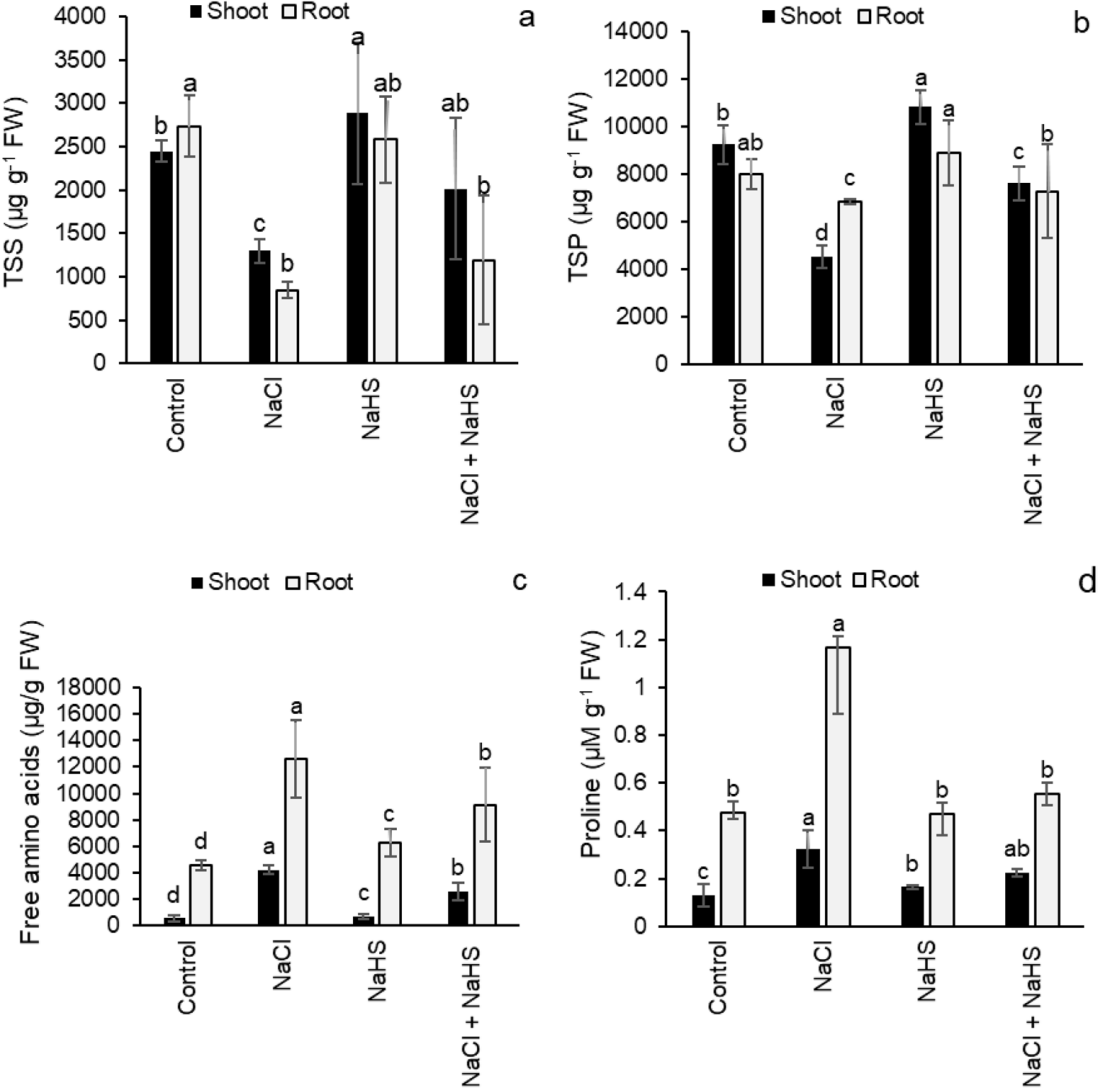
Effect of NaHS pretreatment in the presence and absence of 150 mM NaCl on (**a**) total soluble sugar (TSS) content, (**b**) total soluble protein (TSP) content, (**c**) free amino acids content, and (**d**) proline content of sunflower seedlings. Each value is the mean ± SD of three replicates. Bars with different letters indicate significant differences at *P* ≤ 0.05

NaCl stress significantly reduced the content of TSS by 46.8% and 69.2% in the shoots and roots, respectively, relative to their controls, whereas H_2_S priming significantly enhanced their contents by 54.8% in the shoots and by 41.2% in the roots of salinity-stressed seedlings relative to those received NaCl treatment only (Fig. 7a). Also, NaCl treatment significantly reduced the TSP contents by 51.2% and 14.5% in the shoots and roots, respectively, relative to their controls (Fig. 7b), while H_2_S priming in the presence of NaCl stress enhanced the TSP contents by 68.7% and 6.5% in the shoots and roots, respectively, compared with those of seedlings treated only with NaCl (Fig. 7b). Relative to their controls, the contents of free amino acids and proline were increased by 641.7% and 151.8% in the shoots and by 175% and 146% in the roots, respectively, in response to NaCl stress. H_2_S priming significantly reduced these contents compared with the seedlings receiving only NaCl stress (Fig. 7c, d).

## Discussion

Salt stress seriously affects crop growth and productivity, and changes in growth are typically the first and most noticeable responses of stressed plants. In this study, the fresh weight, dry weight, and leaf area of sunflower seedlings were significantly suppressed under NaCl stress. This salinity-induced decrease in the growth can be attributed to the osmotic action of salinity (i.e., water stress), ionic stress due to excessive ion accumulation, nutritional disequilibrium, reduced photosynthetic capacity (observed in this research), and/or oxidative stress [35–37]. However, salinity-stressed seedlings showed a marked increase in root-to-shoot dry weight ratio which might imply a higher reduction in the shoot dry weight [35] as well as an increase in root biomass production, which may favor the retention of Na^+^ in the root as an adaptive response [6, 35]. We also speculate that the extensive root growth could compensate for their absorption function loss resulting from salinity treatment by dry weight accumulation [38, 39]. The reduction in growth parameters and the higher R/S ratio were also reported in different crop species in response to saline conditions [40–44]. On the other hand, the mitigative effect of H_2_S on salinity-induced growth reduction most probably is interpreted as H_2_S modulation of ion hemostasis, elevation of osmolyte levels (total soluble proteins and total soluble sugars observed in this study), signaling molecule role, and scavenging of ROS under saline imposition [34, 35, 45–48]. Reduced toxic ions and elevated beneficial ones as well as ROS detoxification by H_2_S priming under saline conditions have been reported as adaptive mechanisms for sunflower salinity tolerance [35]. Also, H_2_S priming followed by NaCl stress resulted in a remarkable reduction in the R/S ratio might be explained by the impact of H_2_S in reducing Na^+^ uptake and accumulation in both the root and shoot as previously reported by Younis and Mansour [35].

Stomata are important structures of photosynthetic apparatus, and gas exchange through stomata plays an important role in carbon assimilation. Therefore, abnormalities in the shapes and numbers of stomata can reduce carbon fixation capacity and ultimately disturb the photosynthetic process [49]. In the present study, NaCl treatment caused a significant reduction in stomatal index percentage, stomatal area, and opened stomata, which was accompanied by wrinkling and shrinkage of epidermal walls. These findings agreed with those of Mohamed et al. [50], De Micco et al. [51], and El-Dakak et al. [52], who reported reduced stomatal index percentage and stomatal width in different plant species under salinity stress. Additionally, many factors regulate stomatal mechanics, including ABA, NO, specific ions, and water channels [53, 54]. It has also been shown that ABA accumulation is a cell signaling process in response to salinity stress to help plants survive by inhibiting stomatal opening, transpiration, and plant expansion [53]. In the current work, salinity-induced stomatal closure in leaves of sunflower plants was associated with ABA accumulation and reduced LRWC, which is in accordance with the results of Gupta et al. [55], Gupta and Seth [56], and Niu et al. [54] who demonstrated salinity-induced ABA production was involved in the regulation of stomatal closure to regulate plant internal water balance. However, H_2_S priming improved stomatal opening which was associated with lower ABA content. Our finding is consistent with the results of Lisjak et al. [57, 58], who report H_2_S promotion of stomata to open by reducing ABA accumulation in *Arabidopsis thaliana*. We also propose that stomatal aperture enhancement by H_2_S may increase CO_2_ availability in the mesophyll cells as well as water homeostasis, and thereby stimulate the photosynthesis efficiency observed in this study [30, 34, 45, 59].

Salinity stress affects photosynthesis by perturbing chloroplasts’ ultrastructure and synthesis of pigments [60]. Chloroplasts play a central role in photosynthesis, serving as the primary site for energy conversion and the assimilation of power production [20, 21]. In the current research, the ultrastructure of the chloroplasts was greatly altered under salinity stress showing distorted thylakoid and an unrecognizable grana structure, which might be brought about by salinity-induced oxidative damage [18, 61, 62] and disturbed ion balance in the chloroplasts [63]. Similar impacts of salinity stress on the chloroplast structure were observed in cucumber [64], Arabidopsis [65], potato [66], rice [67], eggplant [17], and faba bean [52]. NaCl stress also caused a significant reduction in photosynthetic pigments (chlorophylls and carotenoids): chlorophyll decline might be due to the inhibition of chlorophyll synthesis due to salinity-reduced levels of iron and magnesium, enhanced activity of chlorophyllase by Na^+^ and Cl^−^ accumulation, and/or photoinhibition or ROS toxicity [18, 68]. The distortion of thylakoid membranes may also lead to chlorophyll loss, as Chuartzman et al. [69] reported. NaCl-induced decline in the carotenoid content could be attributed to the inhibition of carotenoid biosynthesis enzymes’ activity and the downregulation of their transcription levels [70]. Additionally, the decreased value of photosynthetic performance (PI_abs_) and the maximal photochemical efficiency of PSII (F_v_/F_m_) in salinity-stressed sunflower leaves are indicative of the photoinhibition caused by NaCl on the donor side of the PSII [62]. Also, the disappearance of grana observed in this study may cause a reduction in F_m_ and F_v_/F_m_ leading to the downregulation of PSII activity [18, 71]. H_2_S priming, however, enhanced photosynthetic pigment content and preserved chloroplast ultrastructure, suggestive of H_2_S role as a signaling molecule stimulating the chloroplast biogenesis and increasing the quantity of grana lamellae [72], repairing the biomembrane [34], enhancing the antioxidants, and lowering Na^+^ uptake and translocation [35], which ultimately protected the thylakoid membranes and boosted the photosynthetic capacity of NaCl-challenged sunflower plants. Similarly, other published works reported H_2_S promotion of chlorophyll contents and photosynthetic capacity in a variety of plants [73–75]. In the same trend, H_2_S-triggered carotenoid concentration can be explained by the contention that H_2_S upregulated the expression levels of the carotenoid-related genes and thus their biosynthesis enzyme activities [70] as well as enhancing osmoregulators under stress [34]. It is noteworthy that Zhao et al. [70] showed the carotenoid accumulation increase was associated with improved salinity tolerance in carrots as the carotenoids are essential components of the photosynthetic antenna and reaction center complexes, as precursors in signaling pathways, responsible for antioxidant defense, and other protective roles that help plants cope with abiotic stress. Further, H_2_S showed effective recovery potential in enhancing electron transport efficiency and photochemical activity of PSII as reflected by higher F_v_/F_m_ and PI_abs_ values. This H_2_S beneficial effect has also been shown in various crops under stressful conditions [59, 76–78]; altogether reporting that H_2_S plays a key role in activating plant photosynthesis machinery possibly by increasing Mn ion and extrinsic proteins of oxygen-evolving complex, that further improved the electron donation from water to PSII or might have caused the conformational changes in D1 protein, thereby altering the properties of PSII electron acceptor that ultimately improved the PSII activity.

The application of H_2_S elevated the expression level of the small subunit *HaRBCS* gene, which most likely activated ribulose-1,5-bisphosphate carboxylase (Rubisco) and thus enhanced the photosynthesis rate; the finding was reported by other researchers [79, 80]. The Rubisco small subunit is indicated to be a potential engineering target to improve the performance of Rubisco [19], and based on our findings, we recommend it for engineering biology approaches to modulate Rubisco catalysis activating the photosynthesis and hence crop performance under saline conditions. In addition, the accumulation of different osmolytes (soluble sugars, soluble proteins, and carotenoids) in the sunflower seedlings in response to H_2_S priming in the current study, is suggestive of their role in protecting the architecture and functions of the chloroplast membranes, enzymes, and other cell structures against ROS damage and the toxicity of greater concentrations of intracellular toxic ions, as well as acting as osmotic regulators [18, 34, 81–84], which resulted in enhanced photosynthesis, stabilized subcellular structures, and osmotic adjustment, and ultimately the adaptability of sunflower seedlings to NaCl stress. Moreover, NaCl-enhanced burst of proline in sunflower seedlings (more so in roots) might be associated with salinity-induced ABA content observed in this research as ABA has been reported to elevate proline production in various stressed plants under saline conditions [85, 86]. Greater proline, and possibly other free amino acids, in roots relative to shoots observed in this work is most likely attributed to proline higher biosynthesis and active expression of proline transporters reported in roots than in shoots under stress conditions [87]. Salinity-induced proline accumulation was significantly reduced by H_2_S priming, which was previously found by other investigators in crop plants under salinity stress [45, 83, 88, 89]. It seems that the beneficial proline functions in response to saline stress were compensated by the accumulation of other organic osmolytes observed in the current research including soluble sugars, soluble proteins, and carotenoids under H_2_S priming.

The TEM results of the mesophyll cells under NaCl stress showed disorganization of the protoplast which is possibly caused by salinity-induced osmotic stress [90]. Additionally, thickened cell walls, partial plasmolysis, and decreased intercellular spaces under salinity treatment imply water loss from the cells triggered by Na^+^ and Cl^−^ accumulation in the apoplast [66]. Further, the observed vesicles under salinity treatment are most probably interpreted by the sequestration of Na^+^ and Cl^−^ into the vacuoles as well as by membrane system damage induced by ROS toxicity forming small vacuolations [66, 91]. Also, salinity inhibits the absorption of Ca^2+^, further leading to instability of the cell membranes and cell walls, which might cause such cellular aberrations [35]. The H_2_S priming alleviated these cellular aberrations of the mesophyll cells by H_2_S role in biomembrane repairing [34], ROS scavenging, Na^+^, and Cl^−^ uptake restriction as well as K^+^ and Ca^2+^ content elevation [35], resulting in membrane integrity and osmotic balance maintenance reflected in the elevated value of MSI found in this work. Alamer [84] also reported a similar alleviating impact on the MSI by H_2_S supply under salinity stress in wheat.

## Conclusion

As maintaining the structural and functional organization of chloroplasts is vital for the dynamics and flexibility of the photosynthetic apparatus, H_2_S priming effectively modulated the photosynthetic machinery of sunflower under salinity stress as evidenced by stomatal (density and aperture) and non-stomatal (F_v_/F_m_, FI_abs_, Rubisco small subunit overexpression, photosynthetic pigment elevation, and organic osmolyte accumulation) effectors, which implied the potential use of the priming strategy with H_2_S to increase the photosynthetic performance and efficiency and to preserve the chloroplast structure thereby enhancing sunflower tolerance and expectedly its productivity under saline conditions.

## Materials and methods

### Plant growth conditions and stress imposition

Sunflower (*Helianthus annuus* L.) seeds of cultivar ‘Sakha 53’ were obtained from the Agricultural Research Center, Giza, Egypt, and kept in the dark at 4 °C. The seeds were surface sterilized by immersion in 1% (w/v) sodium hypochlorite solution for 10 min and then rinsed thoroughly with distilled water. The sterilized sunflower seeds were divided into two groups: the first group was primed with distilled water (control), and the second one was primed with NaHS (Sigma-Aldrich, USA) as the exogenous H_2_S donor at a concentration of 0.5 mM at room temperature (25 ± 2 °C). Both control and H_2_S-treated seeds were kept in their respective medium for 2 h. The H_2_S concentration (0.5 mM) was adopted based on our previous work [35] that performed under the same growth conditions and exhibited the best results in improving sunflower salinity tolerance. The seeds were then dried on filter paper for approximately 24 h before germination. The seeds were then sown in plastic pots (diameter 15 cm, height 30 cm) containing 1.5 Kg of sieved air-dried clay soil and peat moss (peat moss: clay, 1:1 v/v). The experiment was conducted using a completely randomized design in a controlled growth chamber (model V3-DM, Vision Scientific Company, Daejeon-Si, South Korea) which was maintained at 27/18°C Day/night temperatures, a 50% relative humidity, a photosynthetic photon flux density (PPFD) of 400 µmol m^− 2^ s^1^, and a 14-h photoperiod. NaHS-pretreated and non-pretreated seeds were irrigated with tape water until seedling establishment for 14 d, and then they were exposed to two levels of NaCl (0 and 150 mM) in three replications for 7 d. Each replication was composed of 10 plants. The following treatments of sunflower seedlings were therefore established: (1) control, untreated and irrigated with tape water; (2) NaCl stress, untreated and irrigated with 150 mM NaCl solution; (3) NaHS, NaHS-priming and irrigated with tape water; and (4) NaHS + NaCl, NaHS-priming and irrigated with 150 mM NaCl solution. Twenty-one-day-old seedlings were harvested and five plants per treatment were subjected to measuring growth parameters. Fresh leaves for photosynthetic and microscopic studies were taken as described below, while others were rapidly frozen in liquid nitrogen and stored at -80 °C for other physiological and biochemical analyses.

### Determination of growth parameters

Using a ruler, the plant height (cm) was measured as the distance between the terminal bud and the maximal extremity of the pivoting root. Seedlings were immediately weighed to determine their fresh weight (FW, g per seedling), then dried at 60 °C for 96 h to obtain the dry weight (DW, g per seedling) using a balance (TWIII, USA). Root/shoot ratio (R/S) was obtained through the relation:

$${\rm{R}}/{\rm{S}}\,{\rm{ratio = RDW/(LDW}}\,{\rm{ + }}\,{\rm{SDW)}}$$

where RDW is root dry weight, LDW is leaf dry weight, and SDW is stem dry weight. Leaf area (cm^2^) was obtained by the equation:

$${\rm{Leaf}}\,{\rm{area}}\,({\rm{c}}{{\rm{m}}^2}) = x/y$$

where x is the weight (g) of the area covered by the leaf outline on a millimeter graph paper, and y is the weight of one cm^2^ of the same graph paper.

### Stomatal measurements

For stomatal measurements, the first pair of fully expanded leaves were used. A strip of the upper and lower epidermis from the middle portion of the leaf was peeled off and mounted in glycerol after staining with safranin. Stomatal density (the number of stomata per square millimeter of leaf surface) and sizes were recorded at 400 magnifications under the microscope (Optica, Italy) coupled to a clear camera (digital premiere MA88-900, Italy) and submitted to the Image J Analyzer. The area of the stomata was calculated by multiplying its length by width and then with 0.785; a common factor meant for elliptical structure [92]. The stomatal index was calculated as a percent (%) according to Rengifo et al. [93] using the equation:

$$\begin{array}{ccccc}{\rm{Stomatal}}\,{\rm{Index(\% ) = }} & {\rm{number}}\,{\rm{of}}\,{\rm{stomata/(number}}\,{\rm{of }}\,{\rm{stomata}}\\ & {\rm{ + number}}\,{\rm{of}}\,{\rm{epidermal}}\,{\rm{cells) }} \times {\rm{ 100}}\end{array}$$

The presented data were the mean ± SE of measurements of 10 different fields of view of the leaves’ upper (adaxial) and lower (abaxial) surfaces from 10 individual plants.

### Transmission electron microscopy (TEM)

TEM was performed as described previously by Cao et al. [94]. Samples (1 cm^2^) from the middle of the second flag leaves were collected, then sliced, and fixed immediately in 4% glutaraldehyde in 100 mM sodium cacodylate buffer at pH 7.2 overnight. Following glutaraldehyde fixation, samples were post-fixed in 2% osmium tetroxide in the same cacodylate buffer, stained in 1% aqueous uranyl acetate for 8 h, dehydrated in acetone, and embedded in Spurr’s resin [95]. Ultrathin Sect. (300 nm) of the samples were then examined under transmission electron microscope (TEM; JEM 1011, JEOL, Tokyo, Japan) at 80 kV, and photographed for image analysis with an AMT digital image capture system.

### Measurement of photosynthetic parameters

A portable fluorometer (Handy PEA, Hansatech, Norfolk, UK) was used to determine the photosynthetic performance (PI_abs_) and the maximal quantum yield of PSII photochemistry (F_v_/F_m_) as described by Maxwell and Johnson [22]. Chlorophyll a (Chl a), chlorophyll b (Chl b), and carotenoids (Car) were extracted in 80% (v/v) acetone and measured by UV spectrophotometer UNICAM Helios α (Unicam, Cambridge, UK) according to Metzner et al. [96]. The pigment concentration was calculated and expressed in µg g^− 1^ FW, and then total pigment contents were calculated.

### Determination of total soluble sugars (TSS)

Total soluble sugar concentration was measured according to the method described by Xu et al. [97]. Fresh leaves (0.5 g) were ground with a pestle in an icy mortar, and mixed with 5 mL of distilled water, immediately followed by incubating in boiling water for 30 min. After centrifugation at 4,000 × g for 5 min and removal of the supernatant, the pellet was resuspended and reextracted twice. Three supernatants were transferred to a 25-mL volumetric flask and distilled water was added to make up the volume. Afterward, 1 mL of sample extract was added to 3 mL of anthrone reagent and mixed well. After heating the sample at 100 °C for 10 min and cooling down, the TSS content was measured at the absorbance of 620 nm (Spectronic 601, Milton Roy Company, Texas, USA).

### Determination of total soluble proteins

Total soluble protein contents in leaves were determined using a modified method of Bradford [98]. Fresh samples (1 g) were ground in liquid nitrogen and homogenized in Tris-HCl (100 mM, pH 8.0) extraction buffer containing EDTA (1 mM), DTT (5 mM), Triton X-100 (0.02%, v/v), and glycerol (10%, v/v). The resulting homogenates were centrifuged at 17,000×g for 20 min at 4 °C. One mL of sample extract was added to 5 mL of 0.01% (w/v) Coomassie Brilliant Blue G-250 containing 4.7% (w/v) ethanol and 8.5% (w/v) phosphoric acid and mixed well. After incubation at room temperature for 2 min, the absorbance was recorded at 595 nm (Spectronic 601, Milton Roy Company, Texas, USA).

### Determination of total free amino acids

One gram of fresh tissue was ground thoroughly with 20 mL of distilled water. The mixture was then quantitatively transferred to a boiling tube, and maintained at 80 °C for 15 min. The insoluble residue was removed by filtration and the filtrate was made up to a certain volume and used for the estimation of total free amino acids photometrically with the ninhydrin method of Muting and Kaiser [99].

### Determination of proline content

Extraction of proline was performed according to the method of Carillo and Gibbon [100]. The cold extraction process was used by mixing plant material (0.5 g) with a combination of ethanol: water (40:60 v/v). The supernatant was collected after the mixture was left overnight at 4 °C. Proline was quantified by reading absorbance at 520 nm (Spectronic 601, Milton Roy Company, Texas, USA) and using L-proline as the standard.

### Determination of ABA content

ABA was extracted using freshly collected samples according to the method described by Almeida Trapp et al. [101]. The liquid nitrogen-grinded samples (5 g) were shaken for 30 min and centrifuged at 16,000 x g and 4 °C for 5 min. The supernatant was transferred into a new micro-centrifuge tube and dried in speed vac. After drying, 100 µl of MeOH was added to each sample, which was then mixed with a vortex and centrifuged at 16,000 x g and 4 °C for 10 min. The quantity of ABA was determined using high-performance liquid chromatography (HPLC; instrument E-Chrom Tech, LC 1620, USA). The samples were assayed against ABA as internal standards.

### Leaf relative water content determination

The relative water content of plant leaves (LRWC) was measured by the method of Pan et al. [102]. Leaves from selected plants were detached and weighed to obtain their fresh weight (FW), and then soaked in distilled water at room temperature for 24 h, weighed to obtain turgid weight (TW), and then dried in an oven at 80 °C for 48 h to have a constant dry weight (DW). The LRWC was then calculated using the following formula:

$${\rm{LRWC}}\,(\% ) = (FW - {\rm{DW)/(TW}} - {\rm{DW)}} \times {\rm{100}}$$

### Membrane stability index (MSI) determination

The MSI was determined according to Sairam et al. [103]. Leaf samples (200 mg) were immersed in 10 mL of deionized water and divided into 2 sets. One set was kept at 40 °C for 30 min and its conductivity was recorded (C1) using a conductivity meter (HI 8733, Hanna Instruments, Woonsocket, RI, USA). The second set was kept in a boiling water bath (100 °C) for 15 min and its conductivity was also recorded (C2). The MSI was calculated as:

$${\rm{MSI}}\,{\rm{ = }}\,{\rm{[1 - (C1/C2)]}} \times {\rm{100}}$$

### RNA extraction and quantitative real-time PCR (qRT-PCR)

Total RNA was extracted from 30 mg of fresh leaves of each treatment using a Gene JET™ RNA purification Kit (Thermo Fisher Scientific, MA, USA). One µg of total RNA was reverse transcribed into cDNA using the Revert Aid First Strand cDNA Synthesis Kit (Thermo Fisher Scientific, MA, USA). Ribulose-1,5-bisphosphate carboxylase (Rubisco) small subunit *RBCS* gene sequence was previously searched in the National Center for Biotechnology Information (NCBI, www.ncbi.nlm.nih.gov) to design gene-specific primers for qRT-PCR. Primer sequences for the reactions were *HARBCS1*; Y00431 FP: 5′- CATTTGACACGTGGCTCTCC − 3′; RP: 5′- AGGATGTTGTGGCTCTTGGA − 3 and for AAF82805 (ACTIN sequence from sunflower) FP: 5′-AGGGCGGTCTTTCCAAGTAT − 3; RP: 5′-ACATACATGGCGGGAACATT − 3. PCR amplification specificity was verified using melting curve analysis and data were analyzed using the 2^−ΔΔCt^ method [104] after normalized to the expression of each ACTIN gene.

### Statistical analysis

The results were subjected to a one-way analysis of variance (ANOVA) using the software package SPSS 25.0 (IBM, Corp, Armonk, NY, USA). The comparison of the means of different treatments was carried out using Duncan’s multiple range test at a significant level of 5% (*P* ≤ 0.05). All experimental data are expressed as means ± standard deviation (SD) of three replications.

## Data Availability

All data generated or analyzed during this study are included in this published article
